# Chronic SARS-CoV-2, a Cause of Post-acute COVID-19 Sequelae (Long-COVID)?

**DOI:** 10.3389/fmicb.2021.724654

**Published:** 2021-08-02

**Authors:** Jake S. O’Donnell, Keith J. Chappell

**Affiliations:** ^1^School of Chemistry and Molecular Biosciences, The University of Queensland, St Lucia, QLD, Australia; ^2^The Australian Institute for Biotechnology and Nanotechnology, The University of Queensland, St Lucia, QLD, Australia; ^3^Australian Infectious Disease Research Centre, The University of Queensland, St Lucia, QLD, Australia

**Keywords:** COVID-19, gut flora, immunology, chronic, Long-COVID-syndrome

## Abstract

Severe acute respiratory syndrome coronavirus 2 (SARS-CoV-2) is the cause coronavirus disease 2019 (COVID-19). Most individuals recover from SARS-CoV-2 infection, however, many continue to experience a cluster of persistent symptoms for months following resolution of acute disease; a syndrome that has been named Long-COVID. While the biological cause, or causes, of Long-COVID have not yet been confirmed, the main proposals have centred around either virus-induced autoimmunity or virus-induced tissue dysfunction. However, an alternative suggestion that a latent chronic infection could be responsible for the symptoms of Long-COVID has received minimal attention despite recent findings that SARS-CoV-2 genetic material and infections are detected in some individuals months following resolution of respiratory disease. Here we discuss literature supporting the possibility that Long-COVID occurs as a result of chronic SARS-CoV-2 infections.

The global response to the severe acute respiratory syndrome coronavirus 2 (SARS-CoV-2) pandemic has rightly focused on limiting viral spread through public health measures, prevention of infection through vaccination, and care of patients suffering from severe acute coronavirus disease 2019 (COVID-19) disease. Increased focus is now being placed on the characterisation and management of post-acute COVID-19 sequelae (Long-COVID), which occurs in an estimated 10% of cases. Recently, a high-dimensional analysis of Long-COVID was performed on more than 73,000 COVID-19 survivors. It found that the symptoms of Long-COVID were numerous and included brain fog, chronic fatigue, shortness of breath, sleep disorders, fevers, gastrointestinal problems, anxiety, and depression, among others; defined and discussed in detail elsewhere (Al-Aly et al., 2021). These were associated with significant morbidity and found to persist for months following resolution of acute disease with no defined duration (Carfì et al., 2020; Al-Aly et al., 2021; Huang et al., 2021). While the aetiology of Long-COVID remains undetermined, speculation is currently focused on two possibilities including, (1) that SARS-CoV-2 infections might promote autoimmunity; an association seen for Epstein Barr Virus and Chikungunya virus (Bastard et al., 2020; Cañas, 2020); and (2) that that the utilisation of ACE2 by SARS-CoV-2 as a cellular entry mechanism might down-regulate ACE2 expression and disrupt its essential roles in cellular and tissue homoeostasis (Ashraf et al., 2021). These possibilities are currently receiving considerable attention, however, a third possibility, that Long-COVID might result from latent, chronic SARS-CoV-2 infections of extra-pulmonary tissues, should also be considered (Figure 1). Here, we discuss the existing literature supporting this idea and highlight potential opportunities for future investigations.

**FIGURE 1:**
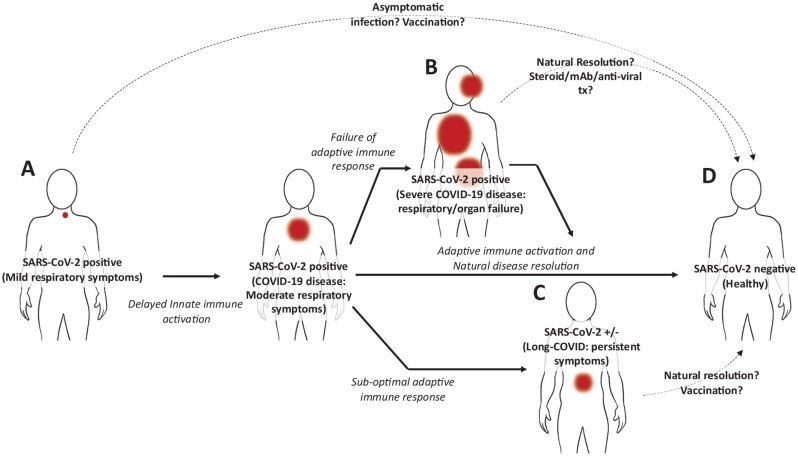
Hypothetical model of SARS-CoV-2 infection and disease progression. **(A)** Following initial infection in the upper pulmonary tract, the SARS-CoV-2 virus evades type I IFNs, delaying innate immune activation. Progressively, respiratory symptoms increase (COVID-19 disease) along with viral load, however, the adaptive immune response against the virus, including T cells and B cells is becoming activated. **(B)** Among individuals unable to induce an effective adaptive immune response to the virus, the innate immune system is aberrantly activated and the virus spreads. In severe cases this can result in pulmonary and organ failure requiring hospitalisation. While this can be fatal, some individuals can recover either untreated or following treatment with steroids, anti-SARS-CoV-2 monoclonal antibodies, and antiviral medications. In these cases it is possible that incomplete viral clearance might also result in Long-COVID. **(C)** In around 10% of individuals who do recover from COVID-19 disease and whose acute symptoms associated with pulmonary disease resolve, a cluster of symptoms can persist including fever, diarrhoea, headaches, and even anxiety and depression. **(D)** Most individuals recover from COVID-19 without lingering symptoms. This likely reflects the induction of an effective adaptive immune response, potentially resulting in immunological memory. Red spots indicate sites and severity of infection. Dotted lines indicate method of disease resolution or treatments.

Entry of SARS-CoV-2 into human tissues requires enzymatic processing of the viral spike protein by TMPRSS2, cathepsin L, and Furin (Hoffmann et al., 2020). This processing enables binding of the spike protein to ACE2 and endocytic entry of the virus into the cell (Hoffmann et al., 2020). The epithelium of the pulmonary tract expresses high levels of these proteins, explaining some of the tropism of SARS-CoV-2 for this tissue. Importantly, these enzymes are co-expressed in extra-pulmonary tissues including the gastrointestinal tract, in the kidneys, heart, and liver (Dong et al., 2020; Beyerstedt et al., 2021). A recent autopsy study looked at a series of 27 patients who died of COVID-19 disease. In addition to the pulmonary tract, SARS-CoV-2 was identified in the kidneys, liver, heart, brain, and gastrointestinal tract; confirming that systemic infections can occur (Braun et al., 2020; Puelles et al., 2020). *In vitro* studies have now shown that gut enterocytes are particularly susceptible to SARS-CoV-2 infection and readily allow for replication of infective virus particles in the intestine (Lamers et al., 2020). One study evaluated gut biopsies collected from a cohort of adults (aged 19–76) taken 4–6 months following an acute SARS-CoV-2 infection. Among 1/3 of participants, SARS-CoV-2 N protein and viral RNA were detected in the intestinal epithelium (Gaebler et al., 2021). Given that these individuals displayed neither persistent pulmonary symptoms nor PCR-detectable infection by nasopharyngeal swab-based testing, these findings support the notion that SARS-CoV-2 infections can persist long-term outside of the pulmonary tract and might explain the source of SARS-CoV-2 detected in waste treatment facilities in the absence of community transmission (Bogler et al., 2020).

Gastrointestinal involvement of COVID-19 is strongly supported by common symptoms including diarrhoea and inflammatory bowel disease with colitis (Zhong et al., 2020). Linking this to the symptoms of Long-COVID, chronic gastrointestinal infections are commonly associated with a variety of immunological disturbances including mucosal inflammation and aberrations in systemic cytokine profiles, which have been reported among individuals with Long-COVID (Doykov et al., 2020). Such chronic infections can manifest in various, and often non-specific symptoms similar to those reported by individuals with Long-COVID: fatigue, fever, diarrhoea, headaches, and even anxiety and depression (Blacklow and Cukor, 1981).

During the initial stages of SARS-CoV-2 infection within the upper respiratory tract, viral replication occurs relatively uninterrupted. This allows for virus to spread into the smaller airways and, although the mechanisms of extrapulmonary spread remain uncharacterised, presumably into other tissues (Gupta et al., 2020). It is understood, however, that the reported high level of viral replication is due in part to the innate ability of SARS-CoV-2 to evade and attenuate the type I Interferon (IFN) pathway (Lee and Shin, 2020). Type I IFNs are critical for antiviral immune responses. In addition to playing a critical role in innate (NK cell, Dendritic Cell, etc.) and adaptive immune cell (T and B cell) activation, they can upregulate class I MHC, induce NK stress ligands (MICA/B), and enable expression of chemokines to attract innate and adaptive immune cells (McNab et al., 2015; Crow et al., 2019). An early study in COVID-19 patients reported that type I IFNs were either not detected regardless of disease severity or at lower levels in plasma, mainly derived from patients with severe disease. The consequence of impaired IFN induction is the development of a unique proinflammatory cytokine profile characterised by high levels of IL-6, IL-1B, and TNF (Leisman et al., 2020; Lucas et al., 2020). This promotes the recruitment *en mass* of pathological inflammatory neutrophils and macrophages, which perpetuate higher levels of proinflammatory cytokines, together associated with considerable pathology (Lucas et al., 2020). Despite these effects, most individuals eventually recover from SARS-CoV-2 infections and COVID-19 disease, indicating that innate and adaptive immune responses can be induced even in the context of low type I IFN induction (Chowdhury et al., 2020; Hadjadj et al., 2020). What is not clear, however, is why an immune response capable of clearing a SARS-CoV-2 infection in the pulmonary tract would be unable to clear a gastrointestinal infection.

It is possible that the residual, infected cells in the gut simply reflect the tail-end of the acute infection which may or may not eventually resolve. This situation is seen for other viral infections which eventually establish themselves as chronic infections including the hepatitis C virus (Rosen, 2011). Several factors might influence whether the immune response originally mounted in the pulmonary tract can effectively clear a latent infection in the gut: (1) following contraction, the remaining T cells might be too few in abundance to come into contact with these rare pockets of infected cells in the gut (Schmidt and Varga, 2018); (2) differences in chemokine expression or metabolite abundance in the gut versus the pulmonary tract might limit T cell recruitment or their viability in the tissue itself (Ahlawat et al., 2020); (3) in the context of the gut, T cells might not receive appropriate antigen stimulation to trigger activation (Zheng et al., 2020); (4) or T cell exhaustion might limit effective clearance of infected cells (Brown et al., 2019). Another compelling explanation might be due to microbial dysbiosis between the gut and the lung caused by the SARS-CoV-2 infection itself. The microbial gut-lung axis is extremely important for physiological development of innate and adaptive mucosal immunity (Sencio et al., 2021; Yeoh et al., 2021). Infections of the pulmonary tract including respiratory syncytial virus (RSV) and influenza have been associated with changes in the composition of the gut microbiome (Groves et al., 2020). These include a reduction in the abundance of gut-associated segmented filamentous bacteria which are critical for mucosal secretion of IgA in the gut (critical for viral neutralisation) (Deriu et al., 2016), and *Bacteroides fragilis*, important for secretion of type I IFNs by gut-associated immune cells (critical for antigen presentation, T cell activation, and control of viral spread in the gut) (Ramakrishna et al., 2019). Emerging studies are now beginning to demonstrate that acute SARS-CoV-2 infections can alter the gut microbiome. For instance, COVID-19 disease has been associated with a reduced abundance of immunomodulatory gut flora including *Faecalibacterium prausnitzii*, *Eubacterium rectale*, and bifidobacteria. Whether this occurs in response to pathological immune activation, the disturbed cytokine profile of many individuals with COVID-19, or the virus itself, these changes were associated with increased disease severity and elevated concentrations of inflammatory cytokines, C reactive protein, lactate dehydrogenase, aspartate aminotransferase, and gamma-glutamyl transferase (Yeoh et al., 2021). Although not yet shown for individuals with Long-COVID, a reasonable hypothesis might be that disturbances to the gut microbiome (reduction of immunosupportive species and/or outgrowth of more deleterious species) of individuals with Long-COVID, occurring as a result of the initial respiratory infection, might limit the efficacy of the mucosal and humoral immune response against virus in the gut, allowing for establishment of a chronic infection (Figure 1).

It is interesting that reports of individuals with Long-COVID are emerging for whom symptoms resolve within days of receiving COVID-19 vaccinations (Arnold et al., 2021; Belluck, 2021). While these reports are primarily anecdotal and formal clinical studies are needed to evaluate this phenomenon, given these responses are brisk (occurring within days of vaccination) this supports the hypothesis presented here; vaccination might serve to simply boost the existing, sub-optimal adaptive immune response, clear residual infections, and in turn eliminate the symptoms of Long-COVID. By contrast, if SARS-CoV-2-induced autoimmunity or tissue dysfunction were causes of Long-COVID, it is difficult to envisage why COVID-19 vaccination would provide such an immediate reprieve from associated symptoms. It is possible that Long-COVID is in fact a number of diseases and vaccination-induced resolution is only seen for those individuals with chronic disease, but not for SARS-CoV-2-induced autoimmunity or tissue dysfunction. These are questions that will need to be answered by large cohort studies. Such studies are critical to further investigate Long-COVID, to establish its causes, and develop effective strategies to treat those currently suffering from it.

## Data Availability Statement

The original contributions presented in the study are included in the article/Supplementary Material, further inquiries can be directed to the corresponding author/s.

## Author Contributions

JO’D and KC both conceived of, wrote, and edited the manuscript. JO’D created the figure. Both authors contributed to the article and approved the submitted version.

## Conflict of Interest

The authors declare that the research was conducted in the absence of any commercial or financial relationships that could be construed as a potential conflict of interest.

## Publisher’s Note

All claims expressed in this article are solely those of the authors and do not necessarily represent those of their affiliated organizations, or those of the publisher, the editors and the reviewers. Any product that may be evaluated in this article, or claim that may be made by its manufacturer, is not guaranteed or endorsed by the publisher.
